# Predicting neurodevelopmental outcomes in neonatal hyperbilirubinemia: a multidimensional nomogram integrating biomarkers and neurobehavioral scores

**DOI:** 10.3389/fped.2026.1825966

**Published:** 2026-05-29

**Authors:** Mei Xue, Jiansong Yin, Jun Lv, Jiaping Yu, Jing Wang

**Affiliations:** Department of Neonatology, The Second People’s Hospital of Changzhou, The Third Affiliated Hospital of Nanjing Medical University, Changzhou, China

**Keywords:** bilirubin-to-albumin ratio, hyperbilirubinemia, neonate, neurodevelopment, NSE—neuron-specific enolase, predicting model

## Abstract

**Objective:**

The long-term neurodevelopmental trajectories of neonates with hyperbilirubinemia lacking overt acute symptoms remain largely unknown. This study aimed to evaluate these trajectories and develop a predictive model integrating multidimensional biomarkers with early neurobehavioral screenings to forecast neurodevelopmental impairment at one year of age.

**Methods:**

In this prospective observational study of 234 term neonates, clinical characteristics were recorded at admission, and laboratory results were obtained within the first 24 h. Early Neonatal Behavioral Neurological Assessment (NBNA) was performed, and neurodevelopment was assessed at 12 months of age using the Gesell Developmental Schedules. Key predictors were selected by integrating LASSO regression results with prior knowledge and clinical significance. A multivariable logistic regression model was subsequently constructed to develop a prognostic nomogram, internally validated using bootstrap resampling.

**Results:**

At the 12-month follow-up, 12.8% (30/234) of the infants exhibited neurodevelopmental impairment. Multivariable analysis identified elevated C-reactive protein (CRP, OR = 1.097, 95% CI: 1.026, 1.172), a higher bilirubin-to-albumin ratio (B/A, OR = 2.587, 95% CI: 1.497, 4.471), and increased neuron-specific enolase (NSE, OR = 1.285, 95% CI: 1.092, 1.513) as independent risk factors for adverse outcomes. Higher NBNA scores (OR = 0.749, 95% CI: 0.608, 0.922) served as an independent protective factor. The model yielded an apparent area under the curve (AUC) of 0.813 and an optimism-corrected AUC of 0.797 (95% CI: 0.696–0.902), demonstrating stable discriminative power. Calibration plots showed consistency between predicted and observed probabilities, with a Brier score of 0.09. Decision curve analysis indicated clinical net benefit across a range of threshold probabilities.

**Conclusion:**

Neonates with hyperbilirubinemia may experience subclinical neurodevelopmental impairment. Our model facilitates risk stratification, supporting targeted long-term follow-up and developmental monitoring for high-risk infants.

## Introduction

In the first week of life, neonatal hyperbilirubinemia ranks among the most common clinical conditions, with roughly 60% of term and 80% of preterm infants affected (1–3). While most cases follow a self-limiting physiological course, significantly elevated levels of free unconjugated bilirubin (Bf) pose a notable risk of irreversible central nervous system damage (1, 4, 5). This lipid-soluble fraction can penetrate the immature blood-brain barrier (BBB) and causes neuronal injury (4–6). Acute Bilirubin Encephalopathy (ABE) represents the acute manifestation of severe bilirubin toxicity. Without timely intervention, ABE may progress to a chronic condition marked by lifelong disabilities such as choreoathetoid cerebral palsy or auditory neuropathy (5).

To prevent such permanent injury, research has long prioritized the early identification of ABE. Total bilirubin (TBIL) levels, the bilirubin-albumin (B/A) ratio, and neuroimaging metrics are regarded as useful for early ABE diagnosis (1, 4). Recently, attention has turned to the long-term prognosis of these infants. But existing cohorts tend to have small sample sizes, with a primary focus on populations presenting overt ABE symptoms or requiring exchange transfusion (6–11). These studies emphasize severe outcomes such as mortality and cerebral palsy, while neglecting more subtle neurodevelopmental impairments in the broader group of patients with hyperbilirubinemia.

Considerable uncertainty persists regarding the long-term neurodevelopmental trajectories of neonates in the “subclinical risk zone” who lack overt acute symptoms. The concept of Bilirubin-Induced Neurologic Dysfunction (BIND) and the broader Kernicterus Spectrum Disorder (KSD) underscores that bilirubin neurotoxicity operates as a continuum rather than an “all-or-nothing” phenomenon (12–14). BIND encompasses a range of subtle sequelae—developmental delay, cognitive impairment, and executive dysfunction—that can arise from jaundice less severe than that causing typical kernicterus (2, 12, 14). Notably, neuronal damage can occur even at TSB levels traditionally deemed safe (<342 μmol/L), producing dysfunction that may remain clinically silent in infancy and become apparent in later childhood (15, 16). While preliminary findings linked hyperbilirubinemia to decreased long-term functional assessment scores (17–19), there is still a lack of models in this field for predicting long-term developmental outcomes.

Adopting a prospective cohort design, this research tracked neonates with hyperbilirubinemia through one year to systematically evaluate their neurodevelopmental trajectories. By integrating biochemical, inflammatory, brain injury biomarkers and early neonatal neurobehavioral assessments, we aimed to identify early predictors of neurodevelopmental impairment at 12 months in neonates with hyperbilirubinemia, and to develop a predictive model for the early identification of high-risk populations. This will facilitate the formulation of targeted follow-up plans and intervention strategies to improve long-term quality of life.

## Methods

### Study participants and data collection

This prospective observational study enrolled neonates with hyperbilirubinemia admitted to the Department of Neonatology, the Second People's Hospital of Changzhou, the Third Affiliated Hospital of Nanjing Medical University, between January 2021 and December 2023. Inclusion criteria: (1) diagnosis of neonatal hyperbilirubinemia according to the standards of the Neonatology Group, Pediatric Society of the Chinese Medical Association; (2) gestational age ≥ 37 weeks; (3) birth weight ≥ 2000 g; and (4) signed informed consent from the legal guardian and agreement to complete follow-up at 1 year. Exclusion criteria: (1) congenital neurological malformations, inherited metabolic disorders, intrauterine infection, or chromosomal abnormalities; (2) hypoxic-ischemic encephalopathy (HIE), intracranial hemorrhage, or central nervous system infection; and (3) Severe infection, sepsis; (4) severely incomplete clinical data or loss to follow-up. All laboratory indicators refer to the first peripheral venous blood tests after admission (within 24 h).

### Neurological function assessment (NBNA)

The 20-item Neonatal Behavioral Neurological Assessment (NBNA) was used to evaluate acute neurological function (20, 21). The assessment was performed as early as possible after ruling out the need for urgent intervention (usually within 72 h after admission). The NBNA scale comprises five domains: behavioral capacity (6 items), passive muscle tone (4 items), active muscle tone (4 items), primitive reflexes (3 items), and general response (3 items), with a maximum score of 40. All assessments were performed by neonatologists who received standardized training and passed inter-rater reliability testing. Evaluations were conducted while the infant was awake and quiet between two feeds. NBNA scores were used to reflect neonatal neurobehavioral status.

### Long-term neurodevelopmental outcome (GESELL assessment)

All enrolled infants returned for neurodevelopmental follow-up at corrected (or chronological) age 12 months ± 2 weeks. The Gesell Developmental Schedules (GESELL) were administered by certified pediatricians who were blinded to group assignment and laboratory results. GESELL assesses five functional domains (22): adaptive behavior, gross motor, fine motor, language, and personal-social behavior. Developmental Quotients (DQ) for each domain were calculated as: DQ = (developmental age/corrected age) × 100. Neurodevelopmental impairment was defined as a DQ < 75 in at least one domain.

### Variable selection strategy

Correlation analysis was first performed for all baseline data, clinical manifestations, laboratory indicators, and short-term functional scores; variables with an absolute correlation coefficient |r| > 0.7 were excluded. Collinearity diagnostics were performed for the preselected variables by calculating variance inflation factor (VIF). Severe multicollinearity was defined as VIF > 5 and highly correlated variables were removed based on clinical judgment. Remaining variables were entered into Least Absolute Shrinkage and Selection Operator (LASSO) regression for feature dimensionality reduction, retaining predictors with nonzero coefficients. To ensure stability, a 5 × 5-fold cross-validation was used to determine the optimal lambda. Variables selected by LASSO underwent a second collinearity diagnosis to confirm absence of multicollinearity. Finally, variables entering the multivariable model were comprehensively determined by integrating prior knowledge in neonatal hyperbilirubinemia and clinical accessibility.

### Model construction and evaluation

The finalized variables were incorporated into a binary Logistic regression model to analyze the association between each factor and neurodevelopmental impairment at 1 year of age. Odds ratios (OR) and their 95% confidence intervals (CI) were calculated. Model calibration was assessed using the calibration curve. Model discrimination was evaluated by the area under the receiver operating characteristic (ROC) curve (AUC). Internal validation of the model was performed using the Bootstrap resampling method (1,000 resampling) to evaluate model stability.

### Statistical analysis

All statistical analyses and figures were produced using R software (version 4.5.0, R Foundation for Statistical Computing, Vienna, Austria). Continuous variables were expressed as mean ± standard deviation or median (interquartile range) and compared using Student's *t*-test or Mann–Whitney *U*-test as appropriate. Categorical variables were presented as frequencies and percentages (%) and compared using the Chi-square test or Fisher's exact test. Variables with a missing rate > 20% were excluded from the analysis. Mean or median imputation was used if the missing rate was <5%; multiple imputation (mice package in R) was applied if the missing rate ranged from 5% to 20%.

## Results

### Study population and baseline characteristics

A total of 234 neonates with hyperbilirubinemia were included. At the 12-month follow-up, 204 infants (87.2%) showed no neurodevelopmental impairment (all DQ ≥ 75), while 30 infants (12.8%) had neurodevelopmental impairment (at least one domain DQ < 75). There were no significant differences in gestational age, birth weight, mode of delivery, feeding method, age at jaundice onset (postnatal day), pre-admission disease duration, or ABO blood type between tow groups (*P* > 0.05). Compared with the normal-development group (ND group), the neurodevelopmental impairment group (NDI group) had a higher proportion of males (73.3% vs. 51.5%, *P* = 0.032) and a longer hospital stay (median 4.5 days vs. 4.0 days, *P* = 0.003).

Laboratory testing showed that C-reactive protein (CRP), white blood cell count (WBC), total bilirubin (TBIL), conjugated bilirubin (CBIL), bilirubin/albumin ratio (B/A), lactate dehydrogenase (LDH), and neuron-specific enolase (NSE) were significantly higher in the NDI group than in the ND group (*P* < 0.05). Other hematologic, thyroid, hepatic, renal and electrolyte indices did not differ significantly between groups (*P* > 0.05) (Table 1).

**Table 1:** Baseline characteristics and laboratory parameters of the study population.

Characteristic	ND group (*n* = 204)	NDI group (*n* = 30)	*P*-value
Demographic & clinical			
Gestational age, weeks	39.0 (38.0, 39.0)	39.0 (38.0, 40.0)	0.747
Birth weight, g	3,350.0 (3,050.0, 3,600.0)	3,330.0 (3,050.0, 3,650.0)	0.749
Sex, *n* (%)			**0**.**032**
Male	105 (51.5)	22 (73.3)	
Female	99 (48.5)	8 (26.7)	
Mode of delivery, *n* (%)			0.307
Vaginal delivery	130 (63.7)	22 (73.3)	
Cesarean section	74 (36.3)	8 (26.7)	
Feeding method, *n* (%)			0.884
Breastfeeding	48 (23.5)	6 (20.0)	
Formula	10 (4.9)	2 (6.7)	
Mixed	146 (71.6)	22 (73.3)	
Onset age, days	1.50 (1.00, 2.00)	1.50 (1.00, 2.00)	0.931
Length of hospital stay, days	4.00 (3.00, 5.00)	4.50 (3.00, 6.00)	**0**.**003**
Thyroid Function			
TSH, mIU/L	7.49 (5.15, 10.81)	7.43 (4.57, 10.52)	0.630
FT3, pmol/L	5.06 (4.23, 5.75)	5.01 (4.51, 6.15)	0.439
FT4, pmol/L	25.15 (22.77, 27.92)	25.02 (23.62, 27.40)	0.883
Hematology			
CRP, mg/L	0.65 (0.50, 2.63)	0.94 (0.63, 5.35)	**0**.**032**
WBC, ×10⁹/L	9.83 (7.91, 12.06)	11.82 (9.76, 13.31)	**0**.**028**
NEUT%	42.60 (34.35, 54.20)	44.25 (34.30, 49.10)	0.977
LYM%	38.85 (29.65, 48.50)	36.55 (28.40, 44.30)	0.484
BASO%	0.40 (0.30, 0.60)	0.40 (0.30, 0.70)	0.876
EO%	3.60 (2.70, 5.25)	4.35 (2.90, 5.70)	0.337
HCT, %	48.00 (43.35, 52.05)	47.25 (43.90, 53.10)	0.809
RBC, ×10¹²/L	4.71 (4.26, 5.09)	4.58 (4.29, 5.21)	0.859
HGB, g/L	166.00 (153.00, 180.50)	167.00 (151.00, 183.00)	0.643
MCV, fL	102.20 (99.80, 104.55)	101.30 (98.80, 104.40)	0.353
MCH, pg	35.10 (34.30, 36.10)	34.95 (34.20, 35.50)	0.424
MCHC, g/L	344.00 (338.00, 350.00)	345.50 (338.00, 351.00)	0.734
MPV, fL	10.10 (9.60, 10.60)	10.20 (9.60, 10.70)	0.377
PLT, ×10⁹/L	295.50 (247.00, 342.00)	298.00 (263.00, 365.00)	0.839
Biochemistry			
TP, g/L	52.10 (49.80, 55.00)	53.20 (50.40, 55.10)	0.643
ALB, g/L	38.25 (36.70, 40.10)	38.90 (36.50, 40.60)	0.376
GLB, g/L	13.70 (12.10, 15.25)	13.70 (10.80, 15.90)	0.716
A/G ratio	2.80 (2.50, 3.20)	2.85 (2.50, 3.60)	0.469
TBIL, μmol/L	242.40 (214.40, 280.90)	290.15 (236.00, 339.50)	**<0**.**001**
CBIL, μmol/L	0.30 (0.10, 0.90)	4.45 (0.30, 11.35)	**<0**.**001**
B/A ratio	3.76 (3.33, 4.26)	4.54 (3.60, 5.19)	**0**.**002**
ALT, U/L	11.95 (9.00, 15.00)	11.90 (9.60, 14.00)	0.931
AST, U/L	40.00 (31.70, 49.55)	39.70 (32.00, 45.00)	0.746
ALT/AST ratio	0.30 (0.24, 0.39)	0.30 (0.23, 0.35)	0.507
ALP, U/L	164.50 (137.50, 203.00)	168.00 (142.00, 209.00)	0.816
GGT, U/L	128.00 (88.50, 185.50)	124.00 (92.00, 189.00)	0.972
LDH, U/L	451.50 (382.00, 524.00)	509.50 (418.00, 570.00)	**0**.**041**
CHE, U/L	5,596.00 (4,780.00, 6,722.50)	5,791.50 (4,757.00, 6,814.00)	0.684
TBA, μmol/L	12.10 (8.80, 18.30)	10.65 (8.30, 16.30)	0.243
ADA, U/L	13.60 (11.65, 15.30)	14.45 (11.80, 15.50)	0.296
BUN, mmol/L	2.00 (1.20, 2.95)	2.60 (1.60, 3.70)	0.071
UA, μmol/L	159.95 (130.10, 196.80)	175.20 (147.20, 224.70)	0.178
K⁺, mmol/L	4.96 (4.60, 5.29)	4.95 (4.68, 5.25)	0.911
Na⁺, mmol/L	139.30 (137.10, 140.90)	139.50 (137.30, 140.70)	0.734
Cl⁻, mmol/L	108.05 (106.10, 110.00)	107.75 (105.90, 109.80)	0.703
CK, U/L	196.50 (121.50, 294.50)	233.50 (149.00, 291.00)	0.315
CK-MB, U/L	36.95 (30.95, 45.50)	39.65 (32.00, 45.00)	0.777
GLU, mmol/L	4.23 (3.70, 4.77)	4.06 (3.69, 4.84)	0.762
UCr, μmol/L	38.35 (33.30, 45.15)	40.00 (32.60, 47.50)	0.734
NSE, ng/mL	34.92 (27.65, 43.76)	41.69 (36.03, 76.97)	**<0**.**001**

TSH, thyroid-stimulating hormone; FT3, free triiodothyronine; FT4, free thyroxine; CRP, C-reactive protein; WBC, white blood cell count; NEUT%, neutrophil percentage; LYM%, lymphocyte percentage; BASO%, basophil percentage; EO%, eosinophil percentage; HCT, hematocrit; RBC, red blood cell count; HGB, hemoglobin; MCV, mean corpuscular volume; MCH, mean corpuscular hemoglobin; MCHC, mean corpuscular hemoglobin concentration; MPV, mean platelet volume; PLT, platelet count; TP, total protein; ALB, albumin; GLB, globulin; A/G, albumin/globulin ratio; TBIL, total bilirubin; CBIL, conjugated bilirubin; B/A, bilirubin/albumin ratio; ALT, alanine aminotransferase; AST, aspartate aminotransferase; ALP, alkaline phosphatase; GGT, gamma-glutamyl transferase; LDH, lactate dehydrogenase; CHE, cholinesterase; TBA, total bile acids; ADA, adenosine deaminase; BUN, blood urea nitrogen; UA, uric acid; CK, creatine kinase; CK-MB, creatine kinase myocardial band; GLU, glucose; UCr, urinary creatinine; NSE, neuron-specific enolase; NBNA, neonatal behavioral neurological assessment; Bold values indicate statistical significance (*P* < 0.05).

### Neonatal neurobehavioral assessment

There was no significant difference in the postnatal age at which the NBNA was performed between groups (*P* = 0.422). The NBNA total score was significantly lower in the NDI group than in the normal-development group (median 35.5 vs. 38.0, *P* < 0.001). On subscale analyses, scores for behavior, passive muscle tone, active muscle tone, and primitive reflexes were all significantly lower in the NDI group (*P* ≤ 0.005) (Table 2).

**Table 2:** Neonatal behavioral neurological assessment (NBNA) scores by neurodevelopmental outcome.

Characteristic	ND group (*n* = 204)	NDI group (*n* = 30)	*P*-value
NBNA assessment age, day	7.00 (5.00, 9.00)	7.50 (6.00, 9.00)	0.422
Behavior	10.00 (10.00, 11.00)	9.00 (9.00, 11.00)	**0**.**005**
Passive tone	8.00 (8.00, 8.00)	8.00 (7.00, 8.00)	**0**.**005**
Active tone	7.00 (7.00, 8.00)	7.00 (7.00, 7.00)	**0**.**002**
Primary reflexes	6.00 (6.00, 6.00)	6.00 (5.00, 6.00)	**0**.**004**
Total NBNA score	38.00 (36.00, 39.00)	35.50 (35.00, 37.00)	**<0**.**001**

Bold values indicate statistical significance (*P* < 0.05).

### Gesell developmental outcomes

Gesell Developmental Scale assessment demonstrated that DQ scores in all domains were significantly lower in the NDI group compared with the ND group (*P* < 0.001), with the largest difference observed in the language domain (Table 3).

**Table 3:** Gesell developmental scale (GESELL) domain quotients by neurodevelopmental outcome.

Characteristic	ND group (*n* = 204)	NDI group (*n* = 30)	*P*-value
Adaptive behavior DQ	90.50 (88.10, 95.00)	78.00 (75.00, 80.00)	**<0**.**001**
Fine motor DQ	90.30 (88.00, 95.00)	73.00 (70.00, 79.00)	**<0**.**001**
Language DQ	89.00 (85.00, 91.20)	68.00 (58.00, 70.00)	**<0**.**001**
Personal-social DQ	91.30 (88.00, 95.00)	78.00 (72.00, 80.00)	**<0**.**001**
Gross motor DQ	92.00 (89.00, 95.30)	80.00 (78.00, 85.00)	**<0**.**001**

Bold values indicate statistical significance (*P* < 0.05).

### Variable selection

**Figure 1:**
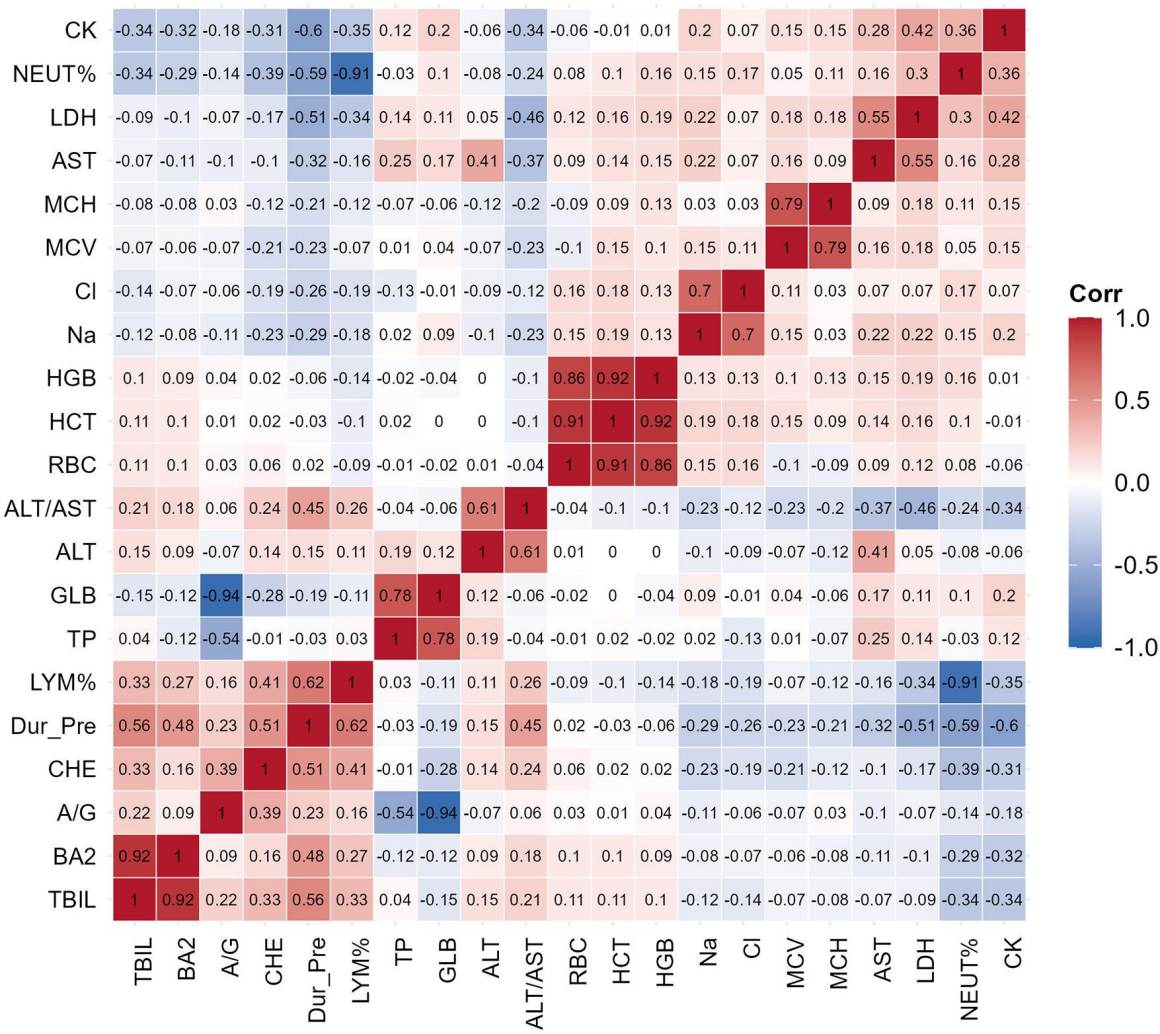
Correlation heatmap of clinical variables. Only 21 variables with high correlation (∣r∣>0.5) were presented.

Correlation analysis identified 21 variable pairs with strong intercorrelation (|r| > 0.5, *P* < 0.05) (Figure 1). Using |r| > 0.7 as a threshold and guided by clinical expertise, 42 variables were preliminarily selected for collinearity diagnostics. The VIF for all variables are all less than 5. Via 5 × 5-fold cross-validation, the LASSO regression model (lambda.min) yielded six predictors with nonzero coefficients: CRP, basophil percentage (BASO%), CBIL, B/A ratio, NSE, and NBNA total score (Supplementary Table S1, Supplementary Figures S1, S2). CBIL primarily reflects hepatobiliary conjugation function, whereas the core neurotoxic moiety in neonatal hyperbilirubinemia is unconjugated (indirect) bilirubin. Because B/A ratio, which comprehensively reflects the bilirubin burden, is already included, CBIL offered no incremental predictive value, nor has prior literature supported its prognostic utility in this setting. BASO% lacks a biologically plausible link to bilirubin metabolism or neurotoxicity, has no established prognostic value in neonatal hyperbilirubinemia, and demonstrates poor measurement stability in neonates. Therefore, to preserve model parsimony and optimize stability given the limited sample size and number of NDI events, we ultimately selected CRP, B/A ratio, NSE, and NBNA total score for inclusion in the multivariable logistic regression model.

### Model development and performance

The results of the multivariate logistic regression analysis are shown in Table 4. CRP (OR = 1.097; 95% CI, 1.026, 1.172; *P* = 0.007), B/A ratio (OR = 2.587; 95% CI, 1.497, 4.471; *P* = 0.001), and NSE (OR = 1.285; 95% CI, 1.092, 1.513; *P* = 0.003) were independent risk factors for NDI, whereas NBNA total score (OR = 0.749; 95% CI, 0.608, 0.922; *P* = 0.006) was an independent protective factor. Specifically, each 10 ng/mL increase in NSE was associated with a 54.4% increase in the odds of developmental impairment; for the other continuous predictors the reported ORs correspond to the change in odds per one-unit increase.

**Table 4:** Multivariable logistic regression analysis of factors associated with neurodevelopmental impairment.

Variable	Odds Ratio (95% CI)	*χ*²	*P*-value
CRP, mg/L	1.097 (1.026, 1.172)	7.33	**0**.**007**
B/A ratio	2.587 (1.497, 4.471)	11.60	**0**.**001**
NSE, ng/mL	1.285 (1.092, 1.513)	9.08	**0**.**003**
NBNA score	0.749 (0.608, 0.922)	7.42	**0**.**006**

CRP, C-reactive protein; B/A, bilirubin/albumin ratio; NSE, neuron-specific enolase; NBNA, neonatal behavioral neurological assessment. For CRP, B/A ratio, and NBNA, ORs represent the change in odds per one-unit increase; for NSE, the OR represents the change per 10 ng/mL.

**Figure 2:**
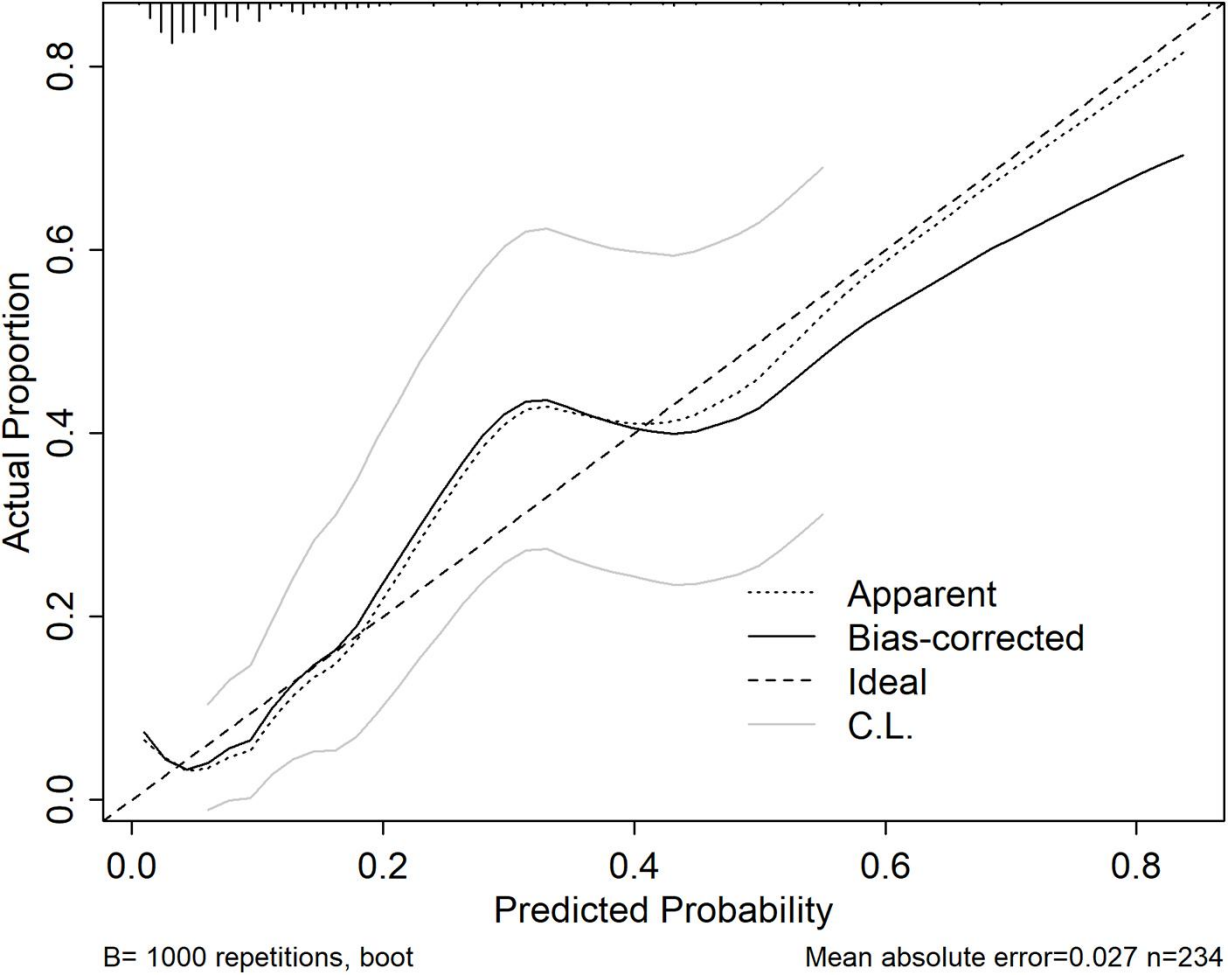
Calibration curves of the multivariable logistic regression model. The bias-corrected line represents the bias-corrected calibration estimated using 1,000 bootstrap resamples, with the gray line indicating the 95% confidence intervals.

Internal validation was performed using 1,000 bootstrap resamples. Apparent AUC was 0.813; optimism-corrected AUC was 0.797 (95% CI, 0.696–0.902) (Table 5). The optimism-corrected calibration intercept was −0.171 (95% CI, −0.969–0.851), the calibration slope was 0.887 (95% CI, 0.445–1.419), and the Brier score was 0.090 (95% CI, 0.063–0.118). The calibration plot is presented in Figure 2. At the cutoff value determined by the Youden index, the model exhibited a sensitivity of 0.833, specificity of 0.804, accuracy of 0.808 (Supplementary Table S2).

**Table 5:** Internal validation and calibration performance of the prediction model.

Performance measure	Original	Optimism-corrected	95% CI (Corrected)
C-index	0.8,134	0.7,974	(0.6,961, 0.9,017)
*R* ^2^	0.3,221	0.2,688	(0.0,715, 0.4,460)
Calibration intercept	0.0,000	−0.1,710	(–0.9,689, 0.8,508)
Calibration slope	1.0,000	0.8,865	(0.4,449, 1.4,190)
Brier score	0.0,821	0.0,898	(0.0,626, 0.1,183)

Internal validation was performed using 1,000 bootstrap resamples.

### Decision curve analysis (DCA) and nomogram

**Figure 3:**
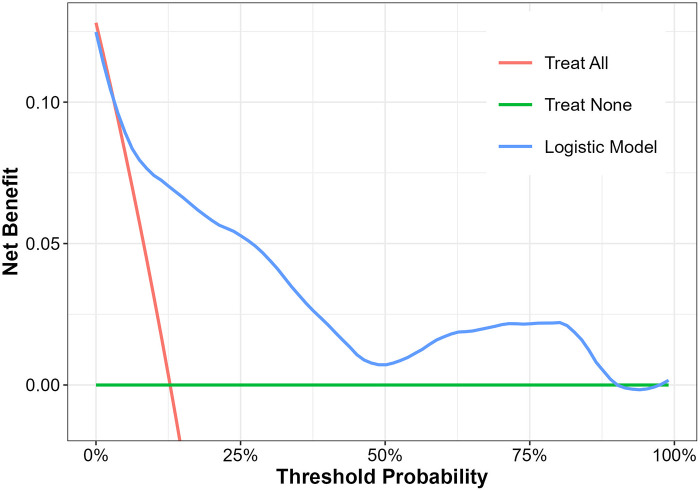
Decision curve analysis (DCA) for the prediction model. The blue line represents the nomogram-based prediction model. The horizontal green line (“Treat None”) assumes no patient receives intervention (net benefit of 0), and the red line (“Treat All”) assumes all patients receive intervention.

DCA demonstrated that the prediction model yielded greater net benefit than the “treat-all” and “treat-none” strategies across a wide range of threshold probabilities (Figure 3).

**Figure 4:**
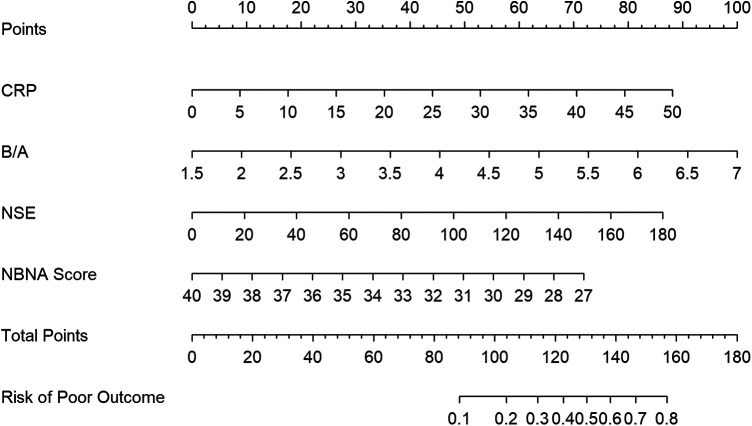
Nomogram for predicting the risk of neurodevelopmental impairment. For example, if a patient has a CRP of 0.7 mg/L (approximately 1 point), a B/A ratio of 4.9 (approximately 64 points), an NSE of 73 ng/mL (approximately 35 points), and an NBNA score of 35 (approximately 31 points), the total score would be 133 points. The total score corresponds to a predicted probability of approximately 0.5, indicating a 50% risk of the child having neurodevelopmental impairment at 1 year of age.

A nomogram based on the logistic regression model was constructed to provide individualized risk estimates (Figure 4). For example, an infant with CRP 0.7 mg/L, B/A ratio 4.9, NSE 73 ng/mL, and NBNA total score 35 would be assigned individual point values from the top point scale: CRP ≈ 1 point, B/A ratio ≈ 64 points, NSE ≈ 35 points, NBNA ≈ 31 points. Summing these yields a total score of 131, which corresponds to a predicted risk of neurodevelopmental impairment at 1 year-old exceeding 50% on the nomogram risk scale.

## Discussion

Tracking the neurodevelopmental trajectories of neonates with hyperbilirubinemia, this prospective cohort study revealed that 12.8% (30/234) of infants exhibited potential neurodevelopmental impairment at 12-month (Gesell DQ < 75 in at least one domain). This suggests that neonates without overt acute brain injury may still suffer neuronal damage. Using feature selection via LASSO regression followed by multivariate logistic regression, we identified the B/A ratio, NSE, and CRP as independent risk factors for adverse outcomes, while the NBNA score served as an independent negative predictor. These findings suggest that the long-term neurodevelopmental outcomes of neonates with hyperbilirubinemia may be associated with bilirubin-related metabolic indicators, systemic inflammatory responses, and the baseline neurobehavioral functional status.

For many years, clinicians have focused primarily on infants with extreme bilirubin levels or overt ABE symptoms. Under the updated KSD framework, however, BIND characterizes the subclinical or mild end of the spectrum, encompassing subtle neurological sequelae such as developmental delay and central nervous system dysfunction. Crucially, BIND may manifest within TSB levels traditionally considered safe (12, 14). Our data and previous evidence (2, 15) suggest that BIND may represent a chronic subclinical exposure that might have been overlooked in the past. The 12.8% incidence of neurodevelopmental impairment in our study population likely reflects infants within the BIND spectrum who were asymptomatic in the neonatal period but later developed minor neurological dysfunction (MND).

The assessment of long-term functional scores explored the possible impact of this subclinical toxicity. We found that children with poor outcomes had lower DQ scores across all functional domains, with the language domain being the most significantly affected. This is consistent with Boskabadi et al. (18), who observed a strong positive correlation between hyperbilirubinemia severity and delays in the language and social domains at a six-month follow-up. These trends also align with data from Ke et al. (17), which showed that severe jaundice is associated with lower scores in cognitive (βadj = −0.44) and fine motor domains (βadj = −0.19$). Slower bilirubin clearance and delayed clinical intervention, as noted by Elmazzahy et al. (19), further raise the likelihood of unfavorable motor and language outcomes. These domain-specific deficits suggest the possibility that high-metabolic brain regions and auditory pathways have a selective vulnerability to bilirubin, even when the degree of injury is insufficient to cause classical cerebral palsy.

Among the identified risk factors, the B/A ratio demonstrated the strongest independent association (OR = 2.587, *P* = 0.001). The B/A ratio serves as a more effective surrogate marker for Bf. An elevated B/A ratio indicates that more lipid-soluble Bf passes through the immature BBB and accumulate preferentially in metabolically active regions including the basal ganglia and auditory brainstem nuclei (2). This accumulation triggers a cascade of neuroinflammation, mitochondrial dysfunction, and oxidative stress, potentially inhibiting neurotransmitter synthesis and eventually contributing to neuronal membrane leakage and apoptosis (2, 6). Kang et al. (8) reported that infants in the adverse outcome group had a significantly higher B/A ratio (10.1 ± 1.7 mg/g) than those in the favorable group (7.5 ± 1.4 mg/g). Our data suggest that a higher B/A ratio may to some extent reflect the severity of subclinical damage, and such damage may subsequently manifest as multi-domain developmental delays.

NSE provided potential evidence of neuronal injury (OR = 1.285 per 10 ng/mL increment, *P* = 0.003), with every 10 ng/mL increase in NSE associated with a 54.4% increase in the risk of neurodevelopmental impairment. As a glycolytic enzyme released during neuronal membrane leakage, elevated NSE may reflect microstructural disruption prior to the onset of clinical symptoms. Zhang et al. (23) demonstrated that NSE is not only significantly elevated in neonates with brain injury (*P* < 0.001) but also negatively correlates with acute neurobehavioral scores (r = −0.785). NSE levels showpromising predictive value for adverse prognosis in neonatal brain injury (AUC = 0.827). Additional evidence indicates that NSE is significantly elevated in hyperbilirubinemic neonates with overt brain injury (24). These data suggest a potential biological link between early structural damage and long-term functional outcomes.

Systemic inflammation may aggravate neurotoxicity by increasing BBB permeability and triggering microglial activation (5, 25). This synergistic effect may explain why neonates with concomitant infections are more susceptible to chronic subclinical exposure, leading to subsequent MND (2, 15). Another study did not find a significant correlation between CRP as well as other inflammatory factors and adverse neurodevelopmental outcomes. A recent systematic review (2) supports a meaningful connection between neonatal CRP and pro-inflammatory cytokine levels and favorable long-term motor and neurodevelopmental courses.

The NBNA score in our model likely represents the integrated neurological response to hyperbilirubinemia (OR = 0.749, *P* = 0.006). Infants with severe hyperbilirubinemia have been found to carry notably lower NBNA scores relative to those with milder jaundice (4). Unlike reactive microscopic laboratory indicators, the NBNA may reflect the macroscopic effects of elevated bilirubin on behavioral ability, muscle tone, and primitive reflexes (20). In this study, higher early NBNA scores were associated with better long-term neurodevelopmental outcomes. Such scores may reflect neuromodulatory capacity and early functional resilience. Early behavioral screening may provide an important functional baseline for identifying children at risk within the BIND spectrum (4, 20).

The primary strengths of this study lie in its prospective cohort design and the integration of multidimensional indicators. By combining biochemical (B/A ratio), structural (NSE), inflammatory (CRP), and functional (NBNA) markers, we constructed a multidimensional predictive framework. Furthermore, the use of LASSO regression allowed for the scientific reduction of over 40 clinical variables, resulting in a preliminary and internally validated model. However, several limitations should be acknowledged to avoid over-interpretation. First, this was a single-center study with a relatively small sample size in the delay group (*n* = 30), and events-per-variable ratio is 7.5:1. This may lead to an overestimation of the diagnostic performance of our predictors. Second, although internal validation was performed, the absence of external validation in an independent cohort limits the generalizability of our findings. Third, we could not entirely rule out the influence of unmeasured confounders, such as genetic susceptibility. Finally, the 12-month follow-up period may be insufficient to detect subtle cognitive or behavioral disorders, such as attention-deficit/hyperactivity disorder or autism spectrum disorder, which typically manifest during school age (2, 11).

## Conclusion

Our study suggests that the B/A ratio, NSE, CRP, and NBNA scores may serve as early predictors of neurodevelopmental impairment at 12 months of age in term neonates with hyperbilirubinemia. The nomogram developed based on these variables offers a visual and individualized tool for the early identification of high-risk infants. We suggest implementing a multimodal screening strategy in clinical practice and enrolling high-risk children in early intervention programs and long-term neurodevelopmental follow-up to ultimately enhance their long-term quality of life.

## Data Availability

The raw data supporting the conclusions of this article will be made available by the authors, without undue reservation.

## References

[1] Sun QS Ye ZX Wu TT Fan QH. Advances in biomarkers of neonatal hyperbilirubinemia. J Biosci Med. (2025) 13:335–47. 10.4236/jbm.2025.138026

[2] Merino-Andrés J Pérez-Nombela S Álvarez-Bueno C Hidalgo-Robles Á Ruiz-Becerro I Fernández-Rego FJ. Neonatal hyperbilirubinemia and repercussions on neurodevelopment: a systematic review. Child Care Health Dev. (2024) 50(1):e13183. 10.1111/cch.13183 37842871

[3] Huang K Wang J Yang Q Zhang G Zheng H Gao Y. Diagnosing acute bilirubin encephalopathy in neonates using MRI-based deep learning model. BMC Pediatr. (2025) 25(1):828. 10.1186/s12887-025-06150-1 41121097 PMC12538867

[4] Liu Q Tang J Deng T Zeng L Zhao H Chen S. Analysis of the diagnostic efficacy of neonatal neurobehavioral assessment scores combined with cranial MRI in brain injury due to severe hyperbilirubinemia in neonates. J Multidiscip Healthc. (2025) 18:6471–8. 10.2147/JMDH.S533256 41084503 PMC12515417

[5] Qian S Kumar P Testai FD. Bilirubin encephalopathy. Curr Neurol Neurosci Rep. (2022) 22(7):343–53. 10.1007/s11910-022-01204-8 35588044

[6] Çolak D Kaynak Türkmen M Özsunar Dayanır Y Aksu H Başak S Tosun AF. Long-term neurodevelopmental outcomes of newborns with high bilirubin levels. Haydarpasa Numune Med J. (2023) 63(2):120–5. 10.14744/hnhj.2021.94759

[7] Helal NF Ghany E Abuelhamd WA Alradem AYA. Characteristics and outcome of newborn admitted with acute bilirubin encephalopathy to a tertiary neonatal intensive care unit. World J Pediatr. (2019) 15(1):42–8. 10.1007/s12519-018-0200-4 30406356

[8] Kang W Yuan X Zhang Y Song J Xu F Liu D. Early prediction of adverse outcomes in infants with acute bilirubin encephalopathy. Ann Clin Transl Neurol. (2020) 7(7):1141–7. 10.1002/acn3.51077 32495505 PMC7359120

[9] McGillivray AJ Polverino J Badawi N Evans NJ. Prospective cohort study of neurodevelopmental outcomes following extreme neonatal hyperbilirubinaemia in Australia. J Paediatr Child Health. (2023) 59(11):1244–50. 10.1111/jpc.16489 37724614

[10] Yi M Lou J Cui R Zhao J. Globus pallidus/putamen T(1)WI signal intensity ratio in grading and predicting prognosis of neonatal acute bilirubin encephalopathy. Front Pediatr. (2023) 11:1192126. 10.3389/fped.2023.1192126 37842026 PMC10570546

[11] Wusthoff CJ Loe IM. Impact of bilirubin-induced neurologic dysfunction on neurodevelopmental outcomes. Semin Fetal Neonatal Med. (2015) 20(1):52–7. 10.1016/j.siny.2014.12.003 25585889 PMC4651619

[12] Bhutani VK Johnson-Hamerman L. The clinical syndrome of bilirubin-induced neurologic dysfunction. Semin Fetal Neonatal Med. (2015) 20(1):6–13. 10.1016/j.siny.2014.12.008 25577653

[13] Johnson L Bhutani VK. The clinical syndrome of bilirubin-induced neurologic dysfunction. Semin Perinatol. (2011) 35(3):101–13. 10.1053/j.semperi.2011.02.003 21641482

[14] Le Pichon JB Riordan SM Watchko J Shapiro SM. The neurological sequelae of neonatal hyperbilirubinemia: definitions, diagnosis and treatment of the kernicterus Spectrum disorders (KSDs). Curr Pediatr Rev. (2017) 13(3):199–209. 10.2174/1573396313666170815100214 28814249

[15] Hegyi T Kleinfeld A. Neonatal hyperbilirubinemia and the role of unbound bilirubin. J Matern Fetal Neonatal Med. (2022) 35(25):9201–7. 10.1080/14767058.2021.2021177 34957902

[16] Xu J Shu D Li Q Wang Y Gu F Zhao X. Clinical analysis of abnormal brainstem auditory evoked potential in neonates with hyperbilirubinemia. Discov Med. (2024) 36(187):1672–7. 10.24976/Discov.Med.202436187.153 39190382

[17] Ke K Chi X Lv H Zhao J Jiang Y Jiang T. Association of breastfeeding and neonatal jaundice with infant neurodevelopment. Am J Prev Med. (2024) 66(4):698–706. 10.1016/j.amepre.2023.11.025 38052381

[18] Boskabadi H Akhondian J Taghipour A Hashemi N Esmaeilzadeh M Nejad SAR. Neonatal hyperbilirubinemia and neurodevelopmental delay assessment at six months of age. Iran J Neonatol. (2023) 14(4):35. 10.22038/ijn.2023.69730.2351

[19] Elmazzahy EA El Din ZE Nessem MA El Tatawy S. Neurodevelopmental outcome at 6 months of age of full-term neonates with hyperbilirubinemia necessitating exchange transfusion. Early Hum Dev. (2024) 190:105969. 10.1016/j.earlhumdev.2024.105969 38341995

[20] Tan Z Wang J Feng J Feng X Huang Y Peng K. Differential item functioning in neonatal behavioral neurological assessment in high-risk full-term infants in NICU based on a machine learning approach. Front Neurosci. (2025) 19:1681152. 10.3389/fnins.2025.1681152 41341261 PMC12669220

[21] Bao XL Yu RJ Li ZS Zhang BL. Twenty-item behavioral neurological assessment for normal newborns in 12 cities of China. Chin Med J. (1991) 104(9):742–6. 10.3760/cma.j.issn.0578-1310.1990.03.117 1935355

[22] Xu X Wang Z Zhang W Guo J Wei W Zhang M. Behavioral observation and assessment protocol for language and social-emotional development study in children aged 0–6: the Chinese baby connectome project. BMC Psychol. (2024) 12(1):533. 10.1186/s40359-024-02031-x 39367488 PMC11451268

[23] Zhang X Dai G Li K. Effectiveness of amplitude-integrated electroencephalography combined with neuron-specific enolase level in predicting neonatal brain injury and prognosis. Am J Transl Res. (2024) 16(10):5398–408. 10.62347/IXFJ7762 39544803 PMC11558382

[24] Cui X Zhou B Wu J Yang D Liu X Wang Y. Changes in amplitude-integrated electroencephalography, neuron-specific enolase, and S100B in neonates with brain injury induced by neonatal hyperbilirubinemia and their significance. Brain Inj. (2021) 35(8):943–8. 10.1080/02699052.2021.1931449 34097553

[25] Silva RF Rodrigues CM Brites D. Bilirubin-induced apoptosis in cultured rat neural cells is aggravated by chenodeoxycholic acid but prevented by ursodeoxycholic acid. J Hepatol. (2001) 34(3):402–8. 10.1016/S0168-8278(01)00015-0 11322201

