# Supplementary material for: Predicting neurodevelopmental outcomes in neonatal hyperbilirubinemia: a multidimensional nomogram integrating biomarkers and neurobehavioral scores
Source: Front Pediatr. 2026 May 29;14:1825966. doi: 10.3389/fped.2026.1825966 (PMC13260602; doi:10.3389/fped.2026.1825966)
Supplement: Supplementary file 1 [file Supplementaryfile1.docx]

Table S1 The non-zero variables selected by LASSO regression

| Index | Coefficient |
| --- | --- |
| Lambda 1.se | 0.0438 |
| CRP | 0.0255 |
| BASO% | 0.0746 |
| CBIL | 0.0023 |
| B/A ratio | 0.3297 |
| NSE, ng/mL | 0.0137 |
| NBNA score | -0.1631 |

Notes: CRP, C-reactive protein; BASO%, basophil percentage; CBIL, conjugated bilirubin; B/A, bilirubin/albumin ratio; NSE, neuron-specific enolase; NBNA, Neonatal Behavioral Neurological Assessment. For NSE, the OR represents the increase in risk per 10 ng/mL increment. Lambda 1.se, lambda value at the 1 standard error of the minimum deviance.

Table S2 Diagnostic Performance of the Prediction Model at the Optimal Cut-off Value

| Parameter | Value | 95% CI |
| --- | --- | --- |
| Youden's index | 1.6373 | - |
| cut-off value | 0.1152 | - |
| Sensitivity | 0.8333 | 0.7000 - 0.9667 |
| Specificity | 0.8039 | 0.7500 - 0.8578 |
| Accuracy | 0.8077 | 0.7564 - 0.8547 |
| Precision | 0.3810 | 0.3158 - 0.4655 |

Notes: Cut-off value was determined by the Youden's index.

*Table S3 Variable missing rate and imputation strategies*

| *Variable* | *Missing cases (n)* | *Missing rate (%)* | *Imputation method* |
| --- | --- | --- | --- |
| TSH | *16* | *6.80%* | *Predictive Mean Matching* |
| FT3 | *16* | *6.80%* | *Predictive Mean Matching* |
| FT4 | *16* | *6.80%* | *Predictive Mean Matching* |
| *CHE* | *21* | *9.00%* | *Predictive Mean Matching* |
| *TBA* | *21* | *9.00%* | *Predictive Mean Matching* |
| *ADA* | *21* | *9.00%* | *Predictive Mean Matching* |
| *NSE* | *5* | *2.10%* | *Median* |

Notes: TSH, thyroid-stimulating hormone; FT3, free triiodothyronine; FT4, free thyroxine; CHE, cholinesterase; TBA, total bile acids; ADA, adenosine deaminase; NSE, neuron-specific enolase.

*Table S4 Highly correlated variables (|r| > 0.7)*

| *variables* | *variables* | *r* |
| --- | --- | --- |
| *GLB* | *A/G* | *-0.936* |
| *TBIL* | *B/A* | *-0.924* |
| *HCT* | *HGB* | *0.919* |
| *HCT* | *RBC* | *0.905* |
| *RBC* | *HGB* | *0.859* |
| *MCV* | *MCH* | *0.788* |
| *NEUT%* | *LYM%* | *-0.908* |
| *TP* | *GLB* | *0.776* |


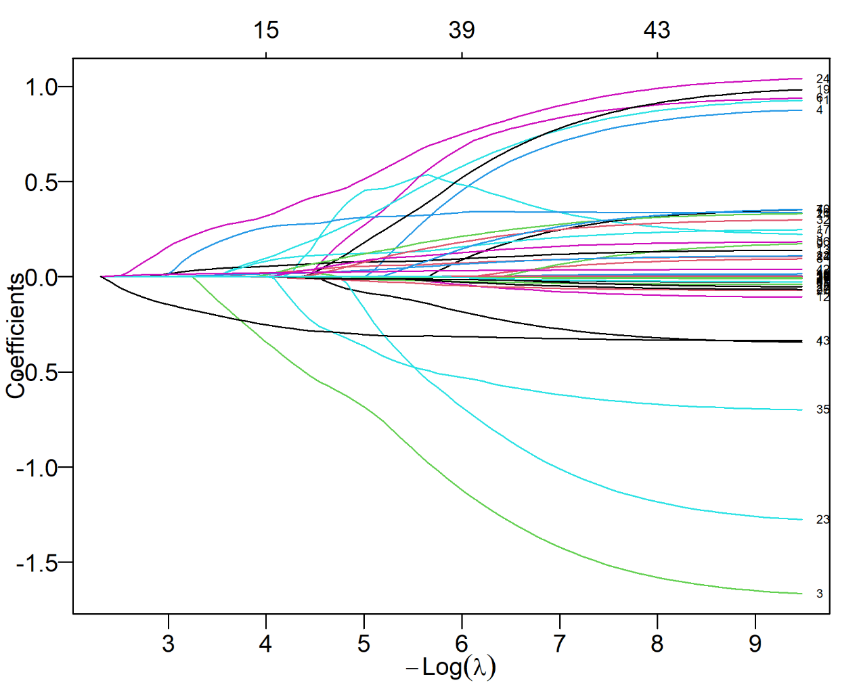


Figure S1 Least Absolute Shrinkage and Selection Operator (LASSO) Regression Path Plot.


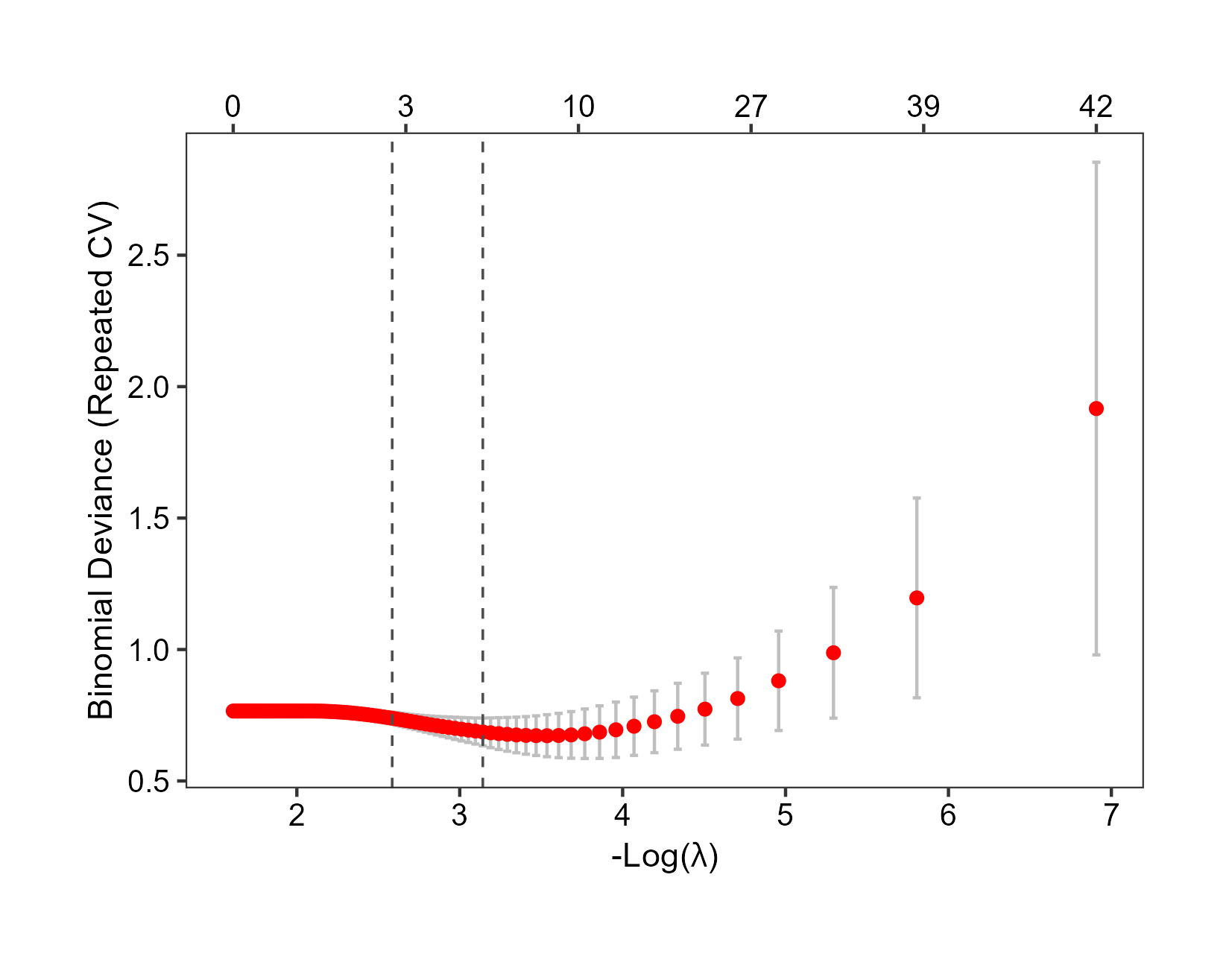


Figure S2 Binomial deviance of the LASSO model as a function of log(λ). The left vertical dashed line corresponds to the λ value at the 1 standard error of the minimum deviance (λ.1se), and the right vertical dashed line corresponds to the λ value at the minimum deviance (λ.min)


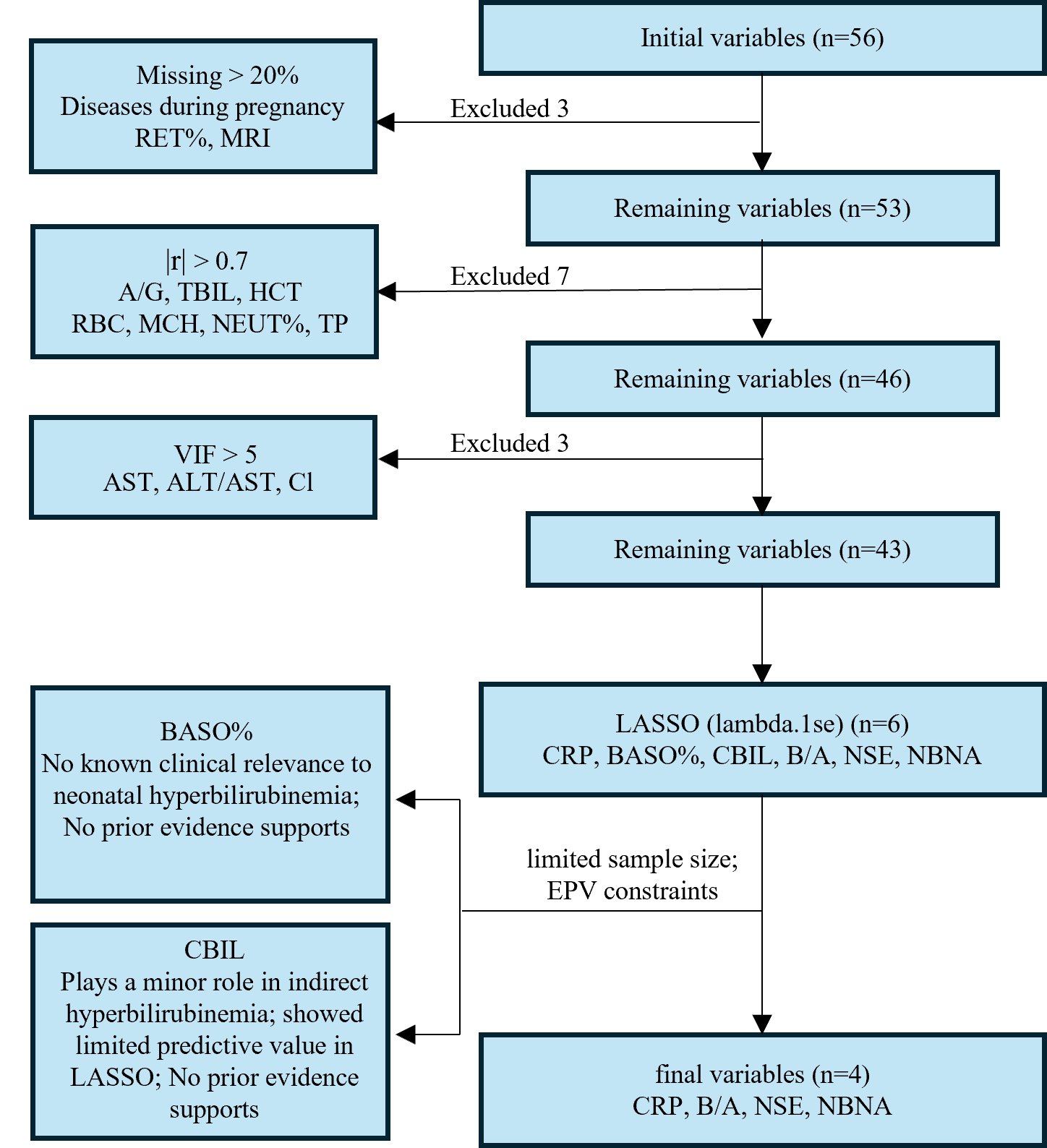


Figure S3 Variable Selection Process
